# Sperm Energy Restriction and Recovery (SER) Alters Epigenetic Marks during the First Cell Cycle of Development in Mice

**DOI:** 10.3390/ijms24010640

**Published:** 2022-12-30

**Authors:** Darya A. Tourzani, Qiangzong Yin, Erica A. Jackson, Oliver J. Rando, Pablo E. Visconti, Maria G. Gervasi

**Affiliations:** 1 Department of Veterinary and Animal Sciences, Integrated Sciences Building, University of Massachusetts, Amherst, MA 01003, USA; 2 Department of Biochemistry and Molecular Biotechnology, University of Massachusetts Chan Medical School, Worcester, MA 01605, USA

**Keywords:** ART, fertilization, in vitro fertilization, pronuclear formation, histone modifications

## Abstract

The sperm energy restriction and recovery (SER) treatment developed in our laboratory was shown to improve fertilization and blastocyst development following in vitro fertilization (IVF) in mice. Here, we investigated the effects of SER on early embryogenesis. Developmental events observed during the first cell cycle indicated that progression through the pronuclear stages of SER-generated embryos is advanced in comparison with control-generated embryos. These findings prompted further analysis of potential effects of SER on pronuclear chromatin dynamics, focusing on the key H3K4me3 and H3K27ac histone modifications. Nearly all the SER-generated embryos displayed H3K4me3 in the male pronuclei at 12 h post-insemination (HPI), while a subset of the control-generated embryos did not. Additionally, SER-generated embryos displayed a more homogenous intensity of H3K27ac at 8 and 12 HPI compared to control embryos. These changes in histone modifications during the first cell cycle were accompanied by differences in gene expression at the two-cell stage; both of these changes in early embryos could potentially play a role in the improved developmental outcomes of these embryos later in development. Our results indicate that sperm incubation conditions have an impact on early embryo development and can be useful for the improvement of assisted reproductive technology outcomes.

## 1. Introduction

Mammalian embryogenesis starts at fertilization with the fusion of the haploid male and female gametes. The early steps of fertilization include the delivery of the sperm-specific phospholipase C zeta (PLCζ) into the metaphase II-arrested oocyte (egg) [1,2,3], which activates the egg by stimulating an increase of intracellular Ca^2+^ and resumption of meiosis [4]. The egg cytoplasm facilitates decondensation and remodeling of the sperm head, allowing for the exposure of the sperm’s nuclear and cytoplasmic contents within the fertilization cone [5,6]. Once the male and female pronuclei are formed, they migrate towards one another in preparation for pronuclear envelope breakdown (NEBD) and syngamy. The timing of NEBD within the first cell cycle of development correlates with early development success in mice [6]. In addition, the sizes of the pronuclei change while migrating. In mice, the male pronucleus (PN) initially exceeds the female PN in size, but as migration occurs the difference in size decreases until the male PN nearly reaches the female PN size at the moment of NEBD [7,8]. Pronuclear size at the time of NEBD has been related to the success of development to the blastocyst stage and birth of offspring [8].

In the first hours after fertilization, the embryonic genome is silent, relying on stored maternal mRNAs for its function. The destruction of accumulated maternal mRNAs and the zygotic initiation of transcription of its own genetic material is known as the maternal-to-zygotic transition (MZT) [9]. Two waves of zygotic gene expression have been described for a successful MZT: minor and major zygotic gene activation (ZGA) [10,11,12]. In mice, the minor ZGA initiates during the S phase of the one-cell stage and ends at the G1 phase of the two-cell stage, followed by the major ZGA occurring during the late two-cell stage (reviewed in [13]). If transcription is prevented during the minor ZGA, further development of the zygote is compromised leading to the halt of development at the two-cell stage [10]. It is well known that transcription is highly dependent on the chromatin structure [14].

The histone proteins are subject to a wide range of covalent modifications, with histone marks including various methylation, acetylation, phosphorylation, and ubiquitination states playing crucial roles in development [13,15,16]. Although the genetic material of the sperm is greatly compacted due to the replacement of most histones with protamines, covalent modifications can modify both the DNA, and the small subset of retained histones, of the sperm genome. Once fertilization has occurred, chromatin status is key to determine transcription during early development. For instance, acetylation of histone 4 lysines 5 and 16 (H4K5/16ac), and histone 3 lysines 9, 14, 18, and 28 (H3K9/14/18/27ac) are histone modifications found in the male PN that are essential for embryo development [17]. In addition, H3K4me3 (tri-methylation) is found in the male PN and is associated with the 5′ ends of actively transcribed genes [18]. Importantly, chromatin dynamics in the early embryo can be modulated by environmental conditions, as, for example, in vitro culture and manipulation of preimplantation embryos can alter epigenetic marks essential for development [19].

Assisted reproductive technologies (ART) have been widely used for the treatment of human infertility and for production and preservation of domestic, laboratory, and wildlife animal species [20,21]. ART involve in vitro manipulation of gametes and embryos, and their success is directly linked to high-quality embryos with the potential to implant and generate progeny [22]. Thus, embryo quality is the major limiting factor for techniques such as in vitro fertilization (IVF) or intracytoplasmic sperm injection (ICSI). Although these techniques are systematically used in the human clinic, both IVF and ICSI present high rates of failure in couples with either female or male subfertility or infertility factors [23,24]. We have recently developed a new method based on sperm incubation in the absence of external nutrients. In these conditions sperm become immotile, and then sperm motility is rescued by the addition of energy metabolites (e.g., glucose and pyruvate) to the incubation medium. This method, named “Sperm Energy restriction and Recovery (SER)” improves sperm functional parameters [25,26]. In particular, it increases sperm motility, fertilization rates, in vitro development to blastocyst, and the number of pups obtained after embryo transfer in mice [25]. In addition, the application of SER enhanced fertilization in mouse models with a male subfertility and infertile phenotype [25,27]. Our previous results highlighted paternal postfertilization effects; however, just how SER impacts early embryonic development remains poorly understood.

Given the well-recognized importance of the events occurring during the first cell cycle in mammalian development, this work investigated whether SER prior to IVF impacts developmental potential via pronuclear dynamics and epigenetic histone modifications. Here, we report an acceleration of the progression through pronuclear stages in the first cell cycle in SER-generated embryos, which was independent of the timing of sperm penetration and cleavage rates. In addition, SER-generated embryos exhibited altered histone modification levels and changes in gene expression, potentially contributing to the increased percentage of SER-embryos that successfully progressed to blastocyst stage. Together, these results suggest that sperm changes occurring prior to fertilization may synchronize critical epigenetic alterations in the zygote and enhance the MZT and development. Our results unveil new roles of postfertilization events on sperm that may lead to improved clinical ART outcomes.

## 2. Results

### 2.1. Timing of Fertilization and Early Embryo Cleavages Are Similar between SER- and Control-Generated Embryos

**Figure 1:**
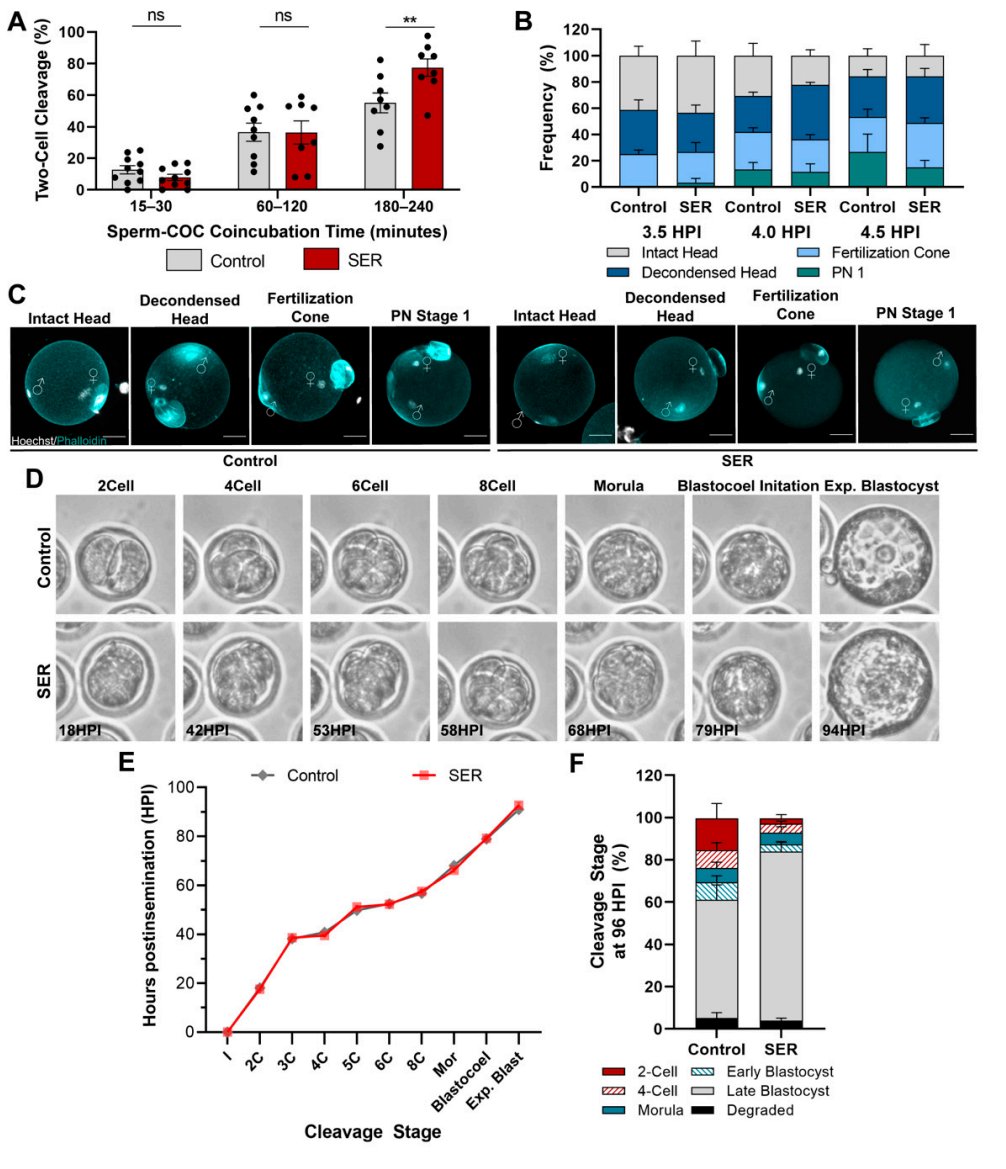
Effects of the SER treatment on early fertilization events. (**A**) Sperm–oocyte penetration time curve assay. Control- and SER-treated sperm were co-incubated with cumulus–oocyte complexes (COCs) for 15–30, 60–120, or 180–240 min before removal and evaluation of fertilization 24 h post-insemination (HPI). N = 6 independent experiments, 20–30 oocytes per time point per condition. Data are mean ± s.e.m. ^ns^ *p* > 0.05, ** *p* < 0.001 (Mixed Model Analysis, Tukey’s). (**B**) Sperm head decondensation comparison at 3.5, 4.0, and 4.5 HPI by 3D-confocal analysis. Analysis categories: intact sperm head, sperm head decondensed, formation of the fertilization cone, and initial formation of pronucleus stage 1 (PN 1). N = 4 independent experiments, 20–30 fertilized embryos per time point per condition. Data are mean ± s.e.m. ^ns^ *p* > 0.05 (Two-way ANOVA). (**C**) Representative confocal microscopy images of control- and SER-fertilized oocytes in each sperm head decondensation category. Cortical actin cytoskeleton (Phalloidin) and DNA (Hoechst) are shown in cyan and grey, respectively. Scale bar: 20 μm. (**D**) Representative time-lapse microscopy images of control- and SER-generated embryos. (**E**) Quantification of time-lapse microscopy analysis of control- and SER-generated embryos. I = insemination, Mor = Morula. N = 4 independent experiments, 10–15 embryos per experiment per condition. Data are mean ± s.e.m. ^ns^ *p* > 0.05 (unpaired *T*-test with Welch’s correction). (**F**) Quantification of control- and SER-generated embryos at 96 HPI. N = 11, control: 247, SER: 352.

SER was previously shown to increase sperm hyperactivated motility and IVF fertilization rates [25]. To determine if the SER-induced increase in fertilization was the result of enhancing the speed of sperm penetration, we coincubated sperm and oocytes for shorter intervals than the standard 4 h in vitro insemination period and evaluated their success of fertilization by visualization of two-cell embryos the following day. Prospective fertilized oocytes were collected from the insemination droplet after 15–30, 60–120, and 180–240 min of coincubation with either control- or SER-treated sperm and incubated overnight in proper conditions. Our results indicated that the SER sperm penetration rate did not differ from the control sperm as the fertilization rates were similar between treatments when sperm–egg coincubation was less than the standard 180–240 min (Figure 1A). Given that fertilization rates were estimated using the percentage of two-cell embryos observed at 18 h post-insemination (HPI), we could not discard the possibility that the increased rate of fertilization in the SER group at 180–240 min resulted from the control-fertilized oocytes being fertilized but failing to cleave to the two-cell stage. To test this possibility, we evaluated the percentage of fused sperm at 4.5 HPI. Consistent with the observed fertilization rates, the rates of sperm–egg fusion were also increased by SER (Figure S1A,B and Supplemental Table S1). These results indicate that the observed differences in fertilization were not due to the control embryos halting development during the first cell cycle. We next evaluated potential differences in the kinetics of early zygotic events such as sperm head decondensation. To accomplish this, we used 3D-confocal analyses of oocytes cocultured with sperm for 3.5, 4.0, and 4.5 HPI. Sperm heads were categorized into four groups: intact sperm head, decondensed sperm head, fertilization cone formation, and initial formation of the male PN. Our analyses revealed no differences between control- and SER-fertilized oocytes with respect to the rate of sperm head decondensation (Figure 1B,C). Altogether, these results indicate that the improvement in fertilization rates is not a simple consequence of the speed of fertilization.

Next, we used time-lapse microscopy to investigate whether SER resulted in changes to the kinetics of embryo progression throughout the preimplantation period (Figure 1D, Videos S1 and S2). These analyses revealed similar cleavage kinetics throughout development of those embryos that successfully develop to blastocyst stage in the control and SER groups (Figure 1E). Nevertheless, further evaluation of the population of embryos at 96 HPI indicated that a significant portion of control-generated embryos halted development at the two- and four-cell stages (Figure 1F and Figure S1C, and supplemental Table S2). These control-generated embryos that failed to progress to blastocyst presented altered cleavage kinetics (Figure S1D). Interestingly, the few SER-generated embryos that failed to progress to blastocyst maintained relatively normal cleavage kinetics before arresting development (Figure S1E). These results suggest that SER treatment is able to coordinate the kinetics of cleavages during early embryo development.

### 2.2. SER-Generated Embryos Are Advanced in Pronuclear Stage Progression

**Figure 2:**
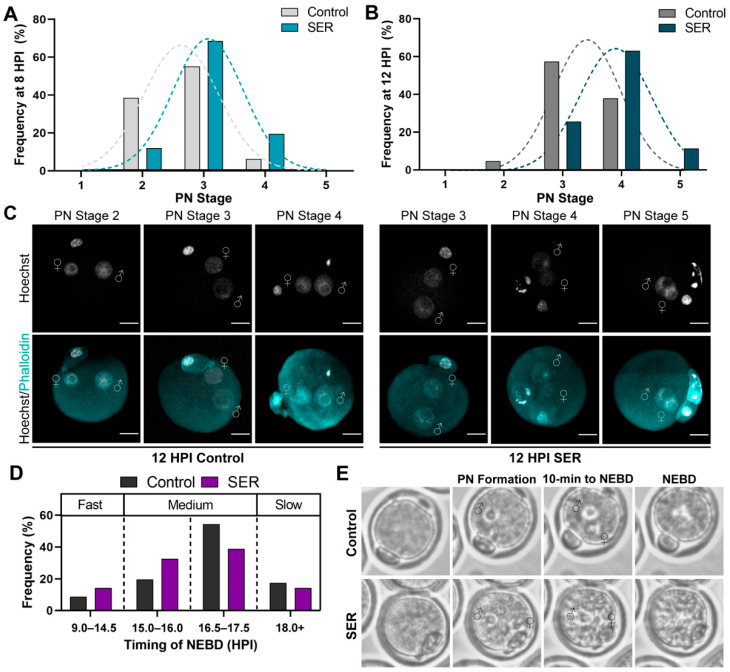
Effects of SER on pronuclear staging and pronuclear envelope breakdown (NEBD). (**A**) Frequency of 8 HPI control- and SER-generated PN stage (1–5) from 3D-confocal analyses. N = 9 independent experiments; number of embryos: control = 145, SER = 133. Chi-square: *p* < 0.0001. (**B**) Frequency of 12 HPI control- and SER-generated PN stage (1–5) from 3D-confocal analyses. N = 10 independent experiments; number of embryos: control = 129, SER = 133. Chi-square: *p* < 0.0001. (**C**) Representative confocal microscopy images of 12 HPI control- and SER-generated embryos. Cortical actin cytoskeleton (Phalloidin) and DNA (Hoechst) are shown in cyan and grey, respectively. Male PN:♂; female PN:♀; and scale bar: 20 μm. (**D**) Quantification of NEBD timing comparison from time-lapse analyses. Analysis categories: fast (9.0–14.5 HPI), medium I (15.0–16.0 HPI), medium II (16.5–17.5 HPI) and slow (18.0+ HPI). N = 4 independent experiments, 10–15 embryos per experiment per condition. (**E**) Representative control- and SER-generated time-lapse images of events leading to NEBD. Male PN:♂, and female PN:♀.

Events occurring within the first cell cycle of development, such as pronuclear migration and syngamy, are key for proper development [6,28]. Therefore, we evaluated the status of the pronuclei during the first hours after insemination. Embryos were collected at 8 and 12 HPI for pronuclear stage classification into five categories according to [17]. Embryonic DNA and cortical actin were stained, imaged by confocal microscopy, and analyzed in three-dimensional (3D) reconstructions to obtain accurate measurements of the distance between male and female pronuclei at each time point for classification into the different pronuclear stages. At both 8 and 12 HPI, the pronuclear stages of SER-generated embryos were significantly advanced compared with control-generated embryos (Figure 2A–C). We next investigated if the accelerated progression though pronuclear staging resulted in changes to the timing of the NEBD. Using time-lapse microscopy, we evaluated the progression of the male and female PN until NEBD, categorizing them into four groups: fast (9.0–14.5 HPI), medium one (15.0–16.0 HPI), medium two (16.5–17.5 HPI), and slow (18.0+ HPI) (Videos S3–S4). The frequency of embryos undergoing NEBD in each group was similar between control- and SER-generated embryos (Figure 2D,E, Table 1). Altogether our results indicate that SER affects pronuclear migration without impacting the timing of NEBD.

### 2.3. SER Results in Differences in Histone Modifications in the Male PN during the First Cell Cycle

**Figure 3:**
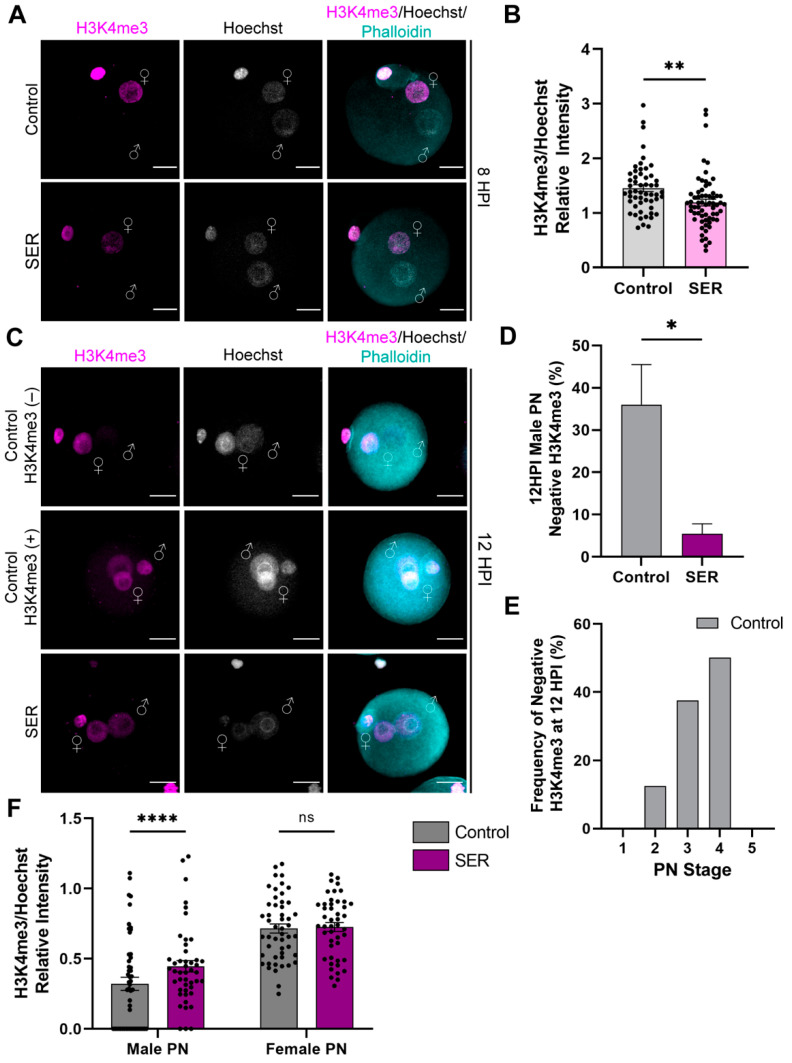
Post-translational histone modification H3K4me3 during pronuclear progression. (**A**) Representative confocal microscopy images of control- and SER-generated embryos at 8 HPI stained for H3K4me3 (magenta). Cortical actin cytoskeleton (Phalloidin) and DNA (Hoechst) are shown in cyan and grey, respectively. Male PN:♂; female PN:♀; and scale bar: 20 μm. (**B**) Quantification of H3K4me3/Hoechst relative intensity of female PN at 8 HPI. N = 5 independent experiments; number of embryos: control = 54, SER = 64. Data are mean ± s.e.m. ** *p* < 0.01 (unpaired *T*-test). (**C**) Representative confocal microscopy images of control- and SER-generated embryos at 12 HPI stained for H3K4me3 (magenta). Control-generated embryos are additionally shown as male PN with H3K4me3 (+) or without H3K4me3 (−) signal. Cortical actin cytoskeleton (Phalloidin) and DNA (Hoechst) are shown in cyan and grey, respectively. Male PN:♂; female PN:♀; and scale bar: 20 μm. (**D**) Quantification of male PN without H3K4me3 signal (negative H3K4me3). N = 6 independent experiments; number of embryos: control = 26 (negative)/65 total, SER: 6 (negative)/81 total. Data are mean ± s.e.m. * *p* < 0.05 (nonparametric test, Mann–Whitney test). (**E**) Frequency of control-generated embryos at each pronuclear stage without H3K4me3 (−) signal in the male PN at 12 HPI. N = 6 independent experiments; 26 embryos. (**F**) Quantification of H3K4me3/Hoechst relative intensity of male and female PN at 12 HPI. N = 5 independent experiments; number of embryos: control = 51, SER = 47. Data are mean ± s.e.m. ^ns^ *p* > 0.05, **** *p* < 0.0001 (unpaired *T*-test).

In mouse embryos, zygotic transcription initiates during the first cell cycle and increases after the first cleavage at the late two-cell stage. Histone modifications are major regulators of transcription during ZGA. To evaluate potential impacts of sperm SER treatment on early embryogenesis, we investigated the status of histone modification markers related to active transcription at different time points during the minor wave of ZGA. First, we analyzed the levels of H3K4me3 by immunofluorescence, normalized to nuclear DNA content. Consistent with existing reports on control embryos, at 8 HPI, only the female PN displayed H3K4me3 signal in both the control- and SER-generated embryos (Figure 3A). Interestingly, the relative intensity of H3K4me3 signal in the female PN decreased in SER-generated embryos (Figure 3B). At 12 HPI, nearly all the SER-generated embryos presented H3K4me3 signal in both the female and male PN, in contrast to control-generated embryos where a large population of embryos exhibited H3K4me3 only in the female PN (Figure 3C,D). We further analyzed whether the lack of H3K4me3 signal in the male PN of some of the control-generated embryos was merely a consequence of a less advanced pronuclear stage. We found no correlation between lack of signal in the male PN and pronuclear stage as even embryos in the advanced PN stage lacked H3K4me3 signal in the male PN (Figure 3E). At 12 HPI, the relative intensity of H3K4me3 was increased in the male PN of SER embryos (Figure 3F). These results indicate that SER results in a more homogeneous population of embryos regarding chromatin status, and that this is independent of the SER effects on pronuclear stage.

**Figure 4:**
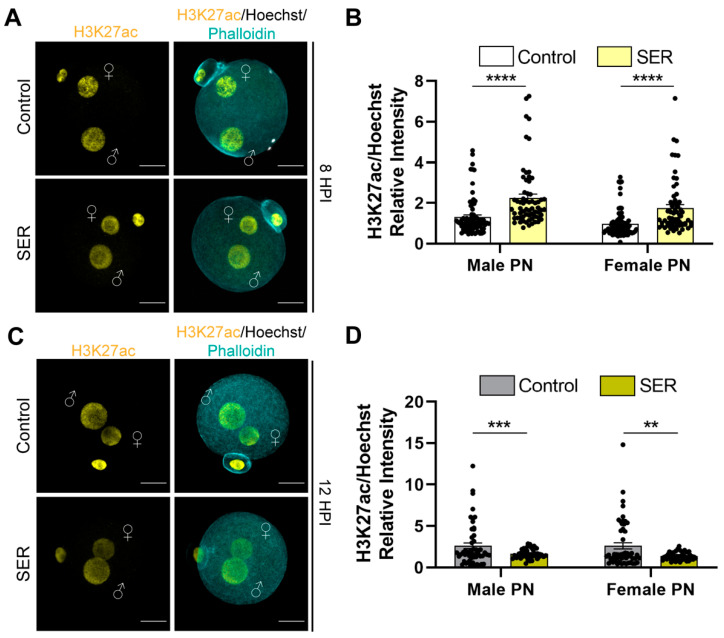
Post-translational histone modification H3K27ac during PN progression. (**A**) Representative confocal microscopy images of control- and SER-generated embryos stained for H3K27ac (yellow) at 8 HPI. Cortical actin cytoskeleton (Phalloidin) and DNA (Hoechst) are shown in cyan and grey, respectively. Male PN:♂; female PN:♀; and scale bar: 20 μm. (**B**) Quantification of H3K27ac/Hoechst relative intensity of male and female PN at 8 HPI. N = 4 independent experiments; number of embryos: control = 75, SER = 64. Data are mean ± s.e.m. **** *p* < 0.0001 (unpaired *T*-test). (**C**) Representative confocal microscopy images of control- and SER-generated embryos stained for H3K27ac (yellow) at 12 HPI. Cortical actin cytoskeleton (Phalloidin) and DNA (Hoechst) are shown in cyan and grey, respectively. Male PN:♂; female PN:♀; and scale bar: 20 μm. (**D**) Quantification of H3K27ac/Hoechst relative intensity of male and female PN at 12 HPI. N = 4 independent experiments; number of embryos: control = 56, SER = 43. Data are mean ± s.e.m. ** *p* < 0.01, *** *p* < 0.001 (unpaired *T*-test).

In addition, we investigated whether the SER treatment had an impact on the acetylation of histone 3 lysine 27 (H3K27ac), another marker associated with active gene transcription found early in both female and male PN. We found differences in the relative intensity of H3K27ac, normalized to nuclear DNA content, in both the female and male PN at 8 HPI (Figure 4A,B). Interestingly, at 12 HPI, the relative intensity of H3K27ac significantly decreased in SER-generated embryos compared to control-generated embryos (Figure 4C,D), indicating possible differences in the transcription dynamics between both groups. A closer evaluation of the relative intensity at 12 HPI revealed two subpopulations within the control-generated embryos (high (>2.5) and mid-to-low (<2.5) H3K27ac) while the SER group again exhibited more homogeneous H3K27ac staining (Figure 4C,D). Given the role of these histone marks in transcription, our findings were consistent with SER effects on male pronuclear histone modifications linked to better outcomes in development. These results suggest potential changes in the regulation of the minor ZGA as a result of the SER treatment prior to fertilization.

### 2.4. Differences in Histone Modifications Continued to Be Observed after the First Cleavage

**Figure 5:**
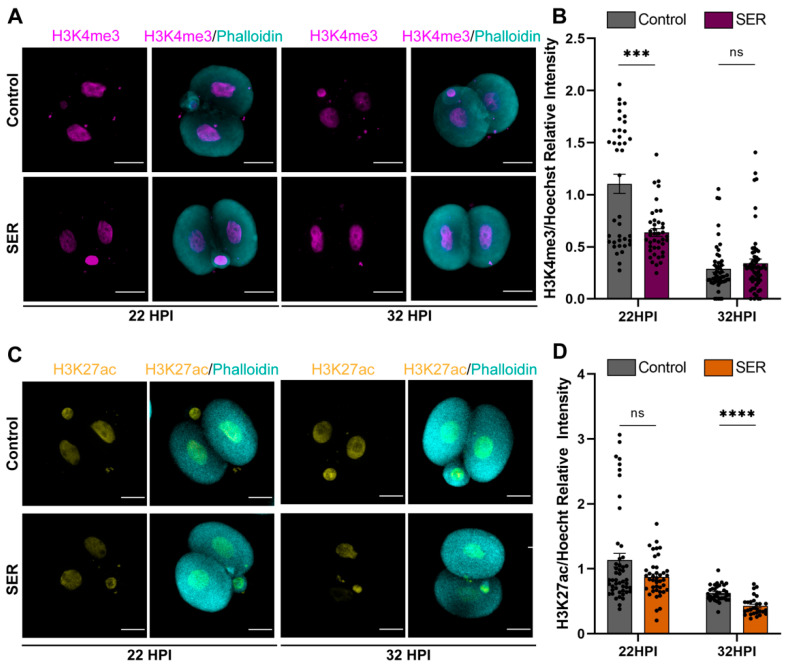
Post-translational histone modifications H3K4me3 and H3K27ac during the timing of ZGA. (**A**) Representative confocal microscopy images of H3K4me3 (magenta) stained control- and SER-generated embryos at 22 and 32 HPI. Cortical actin cytoskeleton (Phalloidin) is shown in cyan. Male PN:♂; female PN:♀; and scale bar: 20 μm. (**B**) Quantification of H3K4me3/Hoechst relative intensity of control- and SER-generated embryos at 22 and 32 HPI. N = 5 independent experiments, 15–20 embryos per time point per condition. Data are mean ± s.e.m. ^ns^ *p* > 0.05, *** *p* < 0.001 (unpaired *T*-test). (**C**) Representative confocal microscopy images H3K27ac (yellow) stained control- and SER-generated embryos at 22 and 32 HPI. Cortical actin cytoskeleton (Phalloidin) is shown in cyan. Male PN:♂; female PN:♀; and scale bar: 20 μm. (**D**) Quantification of H3K27ac/Hoechst relative intensity of control- and SER-generated embryos at 22 and 32 HPI. N = 4 independent experiments, 15–20 embryos per time point per condition. Data are mean ± s.e.m. ^ns^ *p* > 0.05, **** *p* < 0.0001 (unpaired *T*-test).

We continued to investigate whether SER effects on histone modifications sustained through the process of the major ZGA by evaluating two-cell embryos at 22 and 32 HPI. The signal for both H3K4me3 and H3K27ac has been well documented to diminish by the end of the major ZGA in mouse embryos [29]. Therefore, we extended our analysis to include quantifying the relative intensity of H3K4me3 by 3D-confocal microscopy at 22 HPI and 32 HPI (Figure 5A,B). Interestingly, SER-generated embryos exhibited an accelerated loss of H3K4me3 signal at 22 HPI, compared with control embryos where similarly low H3K4me3 levels were observed at 32 HPI. When evaluating H3K27ac intensity, we observed a similar trend at 22 HPI, with two subpopulations of control-generated embryos differing in their levels of H3K27ac (Figure 5C,D). Again, our results indicated that the histone modifications’ kinetics were accelerated in the SER-generated embryos in comparison to the control-generated embryos.

### 2.5. SER Treatment Results in Changes to Gene Expression in Late Two-Cell Embryos

**Figure 6:**
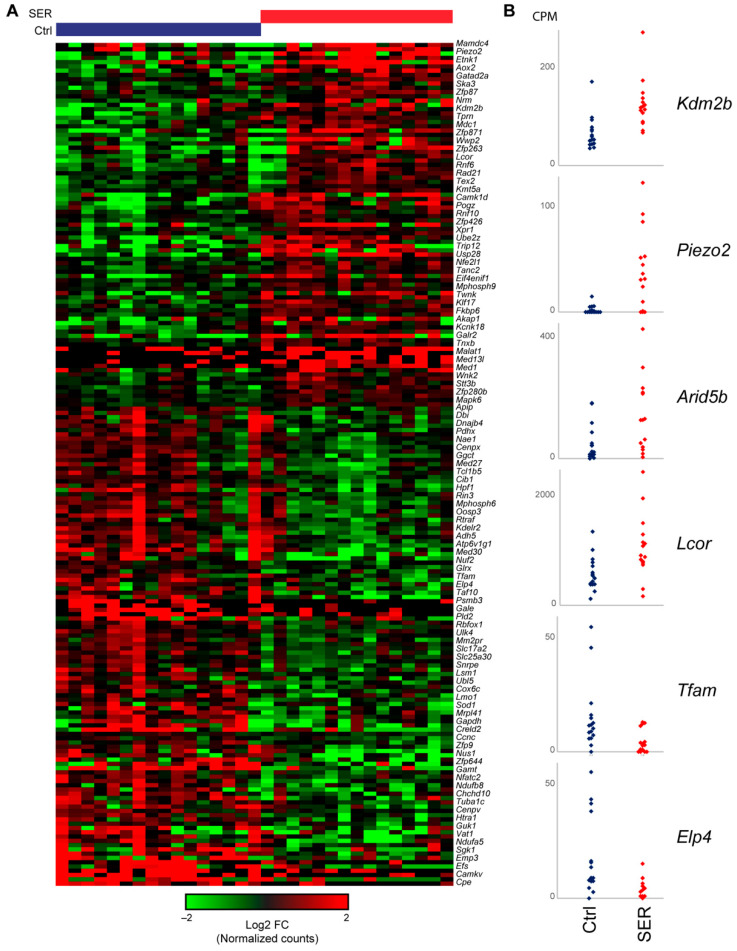
SER treatment impacts gene expression in two-cell stage embryos. (**A**) Heatmap showing differentially expressed genes between control- and SER-generated two-cell embryos. Log2 fold change of expression was calculated for each DEG for all 16 control (blue) and 15 SER (red) two-cell embryos relative to the gene’s median expression across the dataset (all values expressed as log2FC(embryo+10)/(median+10) to suppress expression changes at poorly expressed genes). (**B**) Data for individual two-cell embryos are shown for representative up- or down-regulated genes in SER-generated embryos in comparison to control-generated embryos (Ctrl).

The histone modifications results described above are consistent with SER influencing the process of ZGA. Accordingly, we next sought to determine whether SER treatment altered embryo gene expression profiles shortly after ZGA. Single embryo RNA sequencing (RNA-seq) was performed on three independent batches of control- and SER-generated embryos. After correcting for batch effects, DESeq2 was used to identify 197 differentially expressed genes, as shown in Figure 6A. Data from individual embryos are shown in Figure 6B for several genes of interest to highlight the consistent differences observed between embryo groups. In terms of biological process, differentially expressed genes exhibited relatively little enrichment for significant gene ontology (GO) categories, with upregulated genes enriched for protein ubiquitination, and downregulated genes enriched for various mitochondrial annotations (not shown). However, although not enriched as a class, a number of key genes involved in histone modification and other facets of chromatin organization were among the most significant differentially expressed genes, including not only histone modifying enzymes (Kdm2b, Arid5b, Kat2b, Lcor) but also transcription factors (Nfatc2, Tfam, Zfp3, Stat2) and other chromatin regulators (Rad21). Altered expression of these genes in embryos derived using SER-treated sperm is, of course, of great future interest for understanding the altered epigenetic dynamics of these embryos.

## 3. Discussion

Mammalian embryo development requires the coordination of several morphological, genetic, and epigenetic processes that ensure the formation of a new organism after fertilization. In recent years, in addition to the well-known delivery of the genetic material (DNA) and release of PLCζ into the metaphase II-arrested oocyte triggering its activation, other sperm contributions to early development have been reported [30,31,32]. Our group has developed the sperm energy restriction and recovery (SER) treatment and shown that sperm incubation conditions during in vitro capacitation are relevant for postfertilization events [25]. We have previously shown that SER increases sperm total motility, hyperactivation, fertilization, and preimplantation embryo development rates after IVF in the mouse model [25]. In addition, SER enhanced the implantation potential of the embryos as it increased the number of pups obtained after nonsurgical embryo transfers in mice when compared to control embryos [25]. Moreover, SER prior to ICSI allowed for increased blastocyst development without the need of chemical activation of the egg in the bovine model [25]. Here we tested if the observed effect on fertilization and early embryo development was the result of accelerated sperm penetration and fusion with concomitant variation in cleavages. Our results indicated that SER does not influence the speed of fertilization nor the timing of sequential cleavages of the embryos that develop to blastocyst stage. However, when evaluating the embryos that halt development, SER-generated embryos maintained the average timing of cleavages while the control-generated embryos were highly variable, suggesting that SER could influence the embryonic cell cycle.

Considering the importance of the pronuclear dynamics during the first cell cycle for further development, we aimed to investigate the extent by which SER impacts processes that occur immediately after fertilization during early embryo development. Events such as sperm head decondensation, pronuclear migration, and NEBD are essential for coordinated syngamy of the female and male haploid genomes to form a new individual. In mammals, syngamy is a process prone to errors, and complete synchrony of the male and female genomes is needed for further development [28]. As the male and female pronuclei migrate towards each other, there is a balance between the speed and timing of syngamy that directly relates to the progression to blastocyst stage [6]. Our results indicate that the migration of the pronuclei in SER-generated embryos is faster than in control-generated embryos. Our data are consistent with reports showing that a delayed pronuclear migration kinetics leads to failure of spindle alignment and impairs development [33]. The importance of the first cell cycle kinetics and the synchronous occurrence of NEBD has been also demonstrated for human development [28]. Nevertheless, SER did not affect the timing of NEBD. Altogether, our results indicate that sperm has an impact in pronuclear migration possibly correlating with the observed improvement of preimplantation development. Interestingly, changes in pronuclear formation and improvement of early embryo development after IVF have been reported to depend on the sperm incubation conditions in pigs [34].

In addition to parental genome synchrony, the molecular mechanisms that drive embryo development past the two- and four-cell stage arrive from successful MZT [35]. The contributions of the paternal side to this process are not well understood. Here we show a high efficiency of SER-generated embryos to reach the blastocyst stage, while in contrast a subpopulation of control-generated embryos arrest at the two- and four-cell stage. This finding suggests that not all the control-generated embryos are able to properly undergo MZT. During progression through pronuclear stages, the male pronuclear chromatin undergoes decondensation through the replacement of protamines with histones [36]. In addition, early events of transcription are initiated during the minor ZGA, in which initiation of transcription does not require enhancer activation [37,38,39]. Transcription at the one-cell stage is greater in the male than in the female PN [11]. It has been proposed that a loosened chromatin structure is key for ZGA [40] and the presence of histone post-translational modifications in the chromatin of zygotic pronuclei can directly affect their gene expression [13,17,41]. Here we analyzed the occurrence of H3K4me3 and H3K27ac, markers of active gene expression [42], before the first cleavage. It has been established that H3K4me3 is present in the female PN throughout the course of pronuclear stages, and it appears in the male PN at stage five [43]. Consistent with this report, our results show that H3K4me3 is present in the female PN in all analyzed stages while in the male PN it appears in later stages (12 HPI). Interestingly, nearly all the male pronuclei in the SER-generated embryos present H3K4me3 at 12 HPI, while only a subset of the control-generated embryos does. By analyzing each stage, we ruled out the possibility that this difference in HK4me3 staining was due to differences in pronuclear stages. These data suggest that SER allows for a more homogeneous population of embryos with the ability to increase H3K4me3 in the male PN in a timely fashion. Previously, it has been shown that promoters activated during the major ZGA are modified by H3K4me3 [29,44] suggesting that these differences in H3K4me3 in the male PN could impact ZGA progression.

The occurrence of H3K27ac also increases during the first cell cycle of development [29]. In agreement with the current literature, our results show that H3K27ac is present in both the male and female pronuclei of the embryos during different pronuclear stages and that the levels of H3K27ac taper as development progresses after the first cleavage. Interestingly, again the SER-generated embryos have a more homogenous population regarding H3K27ac intensity at both 8 and 12 HPI in both pronuclei. The heterogeneity found in the control population correlates with the lower development to blastocyst stage observed. In addition, while H3K4me3 relative intensity is increased in SER-generated embryos in comparison to control-generated embryos at 12 HPI, the opposite is observed for H3K27ac. The reasons behind this contrasting H3K4me3 and H3K27ac dynamics remain elusive. We continued to see decreased intensities of both H3K4me3 and H3K27ac in the control- and SER-generated embryos after the first cleavage. Although the dynamics of H3K27ac differ from one report in pigs [45], our results are consistent with the reported levels of H3K27ac during early mouse embryogenesis [46]. The histone modification H3K27ac has been found in promoters of genes transcribed during the major ZGA [29]. Our results suggest that the histone modifications profile of the SER-generated embryos might be influencing gene expression during MZT.

The major wave of transcription occurs after the first cleavage (reviewed in [13]). Our analysis of gene expression after major ZGA indicated that SER induces changes in embryonic gene activation, with a variety of genes being differentially expressed between control- and SER-generated embryos. Genes related to protein ubiquitination were found among the SER upregulated genes. Interestingly, it has been shown that the ubiquitin-proteasome system plays a pivotal role in MZT as it is involved in the degradation of stored maternal proteins in early mouse embryos [47]. The possible link between SER and the clearance of maternal proteins in early embryo development warrants more investigation. In addition, genes involved in histone modifications and chromatin organization are amongst the most upregulated by SER. This is consistent with our premise of SER influencing the epigenetic landscape of the early embryos and modulating development.

Here we showed that transient sperm starvation prior to IVF affects events that occur during the first cell cycle of murine development with a possible impact on development. The mechanism behind these effects remains largely unknown. We have recently shown that incubation in nutrient-free media elevates sperm intracellular calcium concentrations [26]. In addition, we have also shown that a short exposure to the calcium ionophore A23187, increased fertilization and embryo development rates [48]. Despite these works, the extent by which Ca^2+^ in sperm mediates postfertilization SER effects is yet not known. SER may induce molecular changes in the sperm that impact its epigenetic information. One possibility is that the sperm small noncoding RNAs payload changes during the treatment and is directly impacting on the zygote after fertilization. In support of this hypothesis, sperm microRNAs and tRNA fragments are transferred to the oocyte at the moment of fertilization, and these molecules have a role in early embryo development [49,50]. Another possibility is that SER induces changes in sperm chromatin structure that are relevant for embryogenesis. In support of this hypothesis, it was recently described that mouse sperm chromatin presents open regions with transcription factors that are involved in early development already bound to the DNA [32,51]. Considering these possibilities, elucidating the molecular mechanism of SER on sperm and its link with the observed epigenetic changes during the zygote first cell cycle warrants more investigation. Altogether, our results are consistent with the hypothesis that sperm incubation conditions can alter the epigenetic landscape of early embryos and has the potential to be used for assisted reproductive technologies in the clinical setting and animal production.

## 4. Materials and Methods

### 4.1. Animals

Mouse sperm samples were collected from 12–14-week-old C57BL/6J males (Jackson Laboratory, Farmington, CT, USA). Mouse cumulus–oocyte complexes (COCs) were collected from 8–10-week old super ovulated CD1 females (Charles River Laboratories, Wilmington, MA, USA). For superovulation, females were first injected with 7.5–10 IU of pregnant mare serum gonadotropin [PMSG] (Lee BioSolutions, cat # 493-10, Maryland Heights, MO, USA) followed by 7.5–10 IU of human chorionic gonadotrophin [hCG] (Sigma, cat #CG5, St. Louis, MO, USA) 48 h later. COCs were collected 13 h post-hCG injections. Animal care and use of experimental animals were conducted in accordance with specific guidelines and standards dictated by the Office of Laboratory Animal Welfare (OLAW) and approved by the Institutional Animal Use and Care Committee (IACUC), University of Massachusetts Amherst (Protocol #2019-0008).

### 4.2. Media

The medium used for sperm fertilization assay was Toyoda–Yokoyama–Hosi (IVF-TYH) medium. The control sperm capacitation and IVF used a complete medium (IVF-TYH+/+) consisting of 119.37 NaCl, 4.78 KCl, 1.19 NaH_2_PO_4_, 1.19 MgSO_4_·7 H_2_O, 5.56 glucose, 1.71 CaCl_2_·2 H_2_O, 25.1 mM NaHCO_3_^−^, 0.51 Na-pyruvate, 4 mg/mL bovine serum albumin (BSA, Sigma, cat #A0281, St. Louis, MO, USA), 10 µg/mL gentamicin (Sigma, cat #G1272, St. Louis, MO, USA),and 0.0006% phenol red (Sigma, cat #P3632, St. Louis, MO, USA) at pH 7.4 equilibrated with 5% CO_2_. For the SER treatment starvation step, IVF-TYH −/− was used, which was the complete medium mentioned above without the addition of glucose and Na-pyruvate. The medium used for the oocyte collection was Tyrodes’s lactate-HEPES (TL-HEPES), consisting of 114 mM NaCl, 3.22 mM KCl, 2.04 mM CaCl_2_·2 H_2_O, 0.35 mM NaH_2_PO_4_·2H_2_O, 0.49 mM MgCl_2_·6 H_2_O, 2.02 mM NaHCO_3_^−^, 10 mM Lactic acid (sodium salt), and 10.1 mM HEPES at pH 7.4. The medium used for embryo culture was EmbryoMax KSOM mouse embryo media (Sigma, cat #MR-106-D, St. Louis, MO, USA) pre-equilibrated for 1 h at 37 °C in 5% CO_2_ before embryo culture.

### 4.3. SER Treatment

Mice were euthanized by exposure to carbon dioxide (CO_2_). Then, cauda epididymides were dissected and collected into corresponding IVF-TYH media according to the treatment. One cauda was added to IVF-TYH +/+ (control) and the other was added to IVF-TYH −/− (SER). Sperm were recovered in each condition obtained by the “swim-out” method for 10 min, followed by the removal of the cauda tissue. Following this, SER was performed as previously described in [25]. Briefly, the samples were centrifuged for 5 min at 270× *g* at room temperature. For each treatment, supernatant was removed, and 1 mL of the appropriate medium (either IVF-TYH +/+ or −/−) was added to the sperm pellet followed by an additional centrifugation for 5 min at 150× *g* at room temperature. The supernatants were removed, and 1 mL of the corresponding medium was added to each treatment (IVF-TYH +/+ or −/−). The sperm were incubated at 37 °C in 5% CO_2_ until motility of the sperm incubated in the IVF-TYH −/− was lost (between 15 and 45 min). Upon loss of motility, both control and starved sperm were recovered with the addition of 1 mL of IVF-TYH +/+ media (recovery step in which the IVF-TYH −/− sperm are exposed to nutrients glucose and pyruvate) and centrifuged for 5 min at 150× *g* at room temperature. The supernatants were removed, and each tube was resuspended to 1 mL with IVF-TYH +/+ media. Sperm were immediately used for insemination of cumulus–oocyte complexes (COCs) as detailed in the in vitro fertilization section below.

### 4.4. In Vitro Fertilization (IVF) and Embryo Culture

COCs were collected in TL-HEPES 13 h post hCG injection, then washed with IVF-TYH +/+ prior to allocating them in the insemination droplets. Insemination dishes were prepared prior (4, 90 µL droplets of IVF-TYH +/+ covered in mineral oil (Fisher Scientific, cat #0121, Waltham, MA, USA)) and equilibrated overnight at 37 °C in 5% CO_2_. COCs were added to a single droplet in the insemination dish. After collection and treatment of the sperm as described above, each dish was inseminated with approximately 100,000 sperm of either the control- or SER-treated sperm. Insemination dishes were placed back into the incubator and cocultured for 4 h. At 4 h postinsemination (HPI), oocytes were removed from the insemination droplets and washed through the remaining clean droplets of IVF-TYH +/+. Embryo culture dishes were prepared prior (five 50 µL droplets of KSOM covered in mineral oil) and equilibrated overnight at 37 °C in 5% CO_2_. Oocytes from each treatment group were moved into KSOM dishes for embryo culture. Control- and SER-generated embryos were evaluated and collected at 8, 12, 22, and 32 HPI for staining and immunofluorescence, as detailed below or cultured until 96 HPI for blastocyst evaluation.

### 4.5. Sperm–COCs Coincubation Assay

COCs were collected as previously described and separated into six IVF-TYH +/+ insemination dishes per treatment (control or SER). After collection and treatment of the sperm as described above, each dish was inseminated with approximately 100,000 sperm of either control- or SER-treated sperm. Insemination dishes were placed back into the incubator and cocultured for 15, 30, 60, 120, 180, or 240 min before oocytes were removed and washed through a droplet of hyaluronidase (Sigma, cat #H4272, St. Louis, MO, USA) for 2–5 min to eliminate any sperm bound to the surface of the oocyte. Oocytes were then washed through IVF-TYH +/+ and cultured in the incubator at 37 °C in 5% CO_2_ for 20 h. The following day, fertilization was evaluated by the visualization of two-cell cleavage.

### 4.6. Time-Lapse Imaging

In vitro fertilization was performed as described above. Following the 4 h of coculture, oocytes from each treatment group were removed from the insemination droplet and washed through the remaining clean droplets of IVF-TYH +/+. Time-lapse dishes were prepared prior with 30 µL of KSOM covered in mineral oil and equilibrated overnight at 37 °C in 5% CO_2_. Oocytes from each treatment group were moved into time-lapse dishes and imaged every 30 min for 4 days with individual CytoSMART Lux2 Duo devices (CytoSMART, cat #JAC-1008, Eindhoven, Netherlands) while in the incubator at 37 °C in 5% CO_2_.

### 4.7. Sperm Head Decondensation Assay

COCs were collected as described above and separated into six IVF-TYH +/+ insemination dishes as per the treatment previously prepared. After collection and treatment of the sperm as described above, each dish was inseminated with approximately 100,000 sperm of either control- or SER-treated sperm. Insemination dishes were placed back into the incubator and cocultured for 3 h before oocytes were removed and washed through a droplet of hyaluronidase for 2–5 min to eliminate any sperm bound to the surface of the oocyte. Prospective fertilized oocytes were then washed through IVF-TYH +/+ and cultured in the incubator at 37 °C in 5% CO_2_. To evaluate sperm head decondensation, prospective fertilized oocytes were collected and fixed in 4% paraformaldehyde (PFA, Fisher Scientific, cat #50-980-487, Waltham, MA, USA) for 20 min at RT at three different time points: 3.5, 4.0, and 4.5 HPI. Samples were washed with 0.1% Triton X-100 (Sigma, cat #93443, St. Louis, MO, USA) in filtered PBS then permeabilized with 0.5% Triton X-100 in filtered PBS for 20 min at RT, followed by additional three washes with 0.1% Triton X-100 in filtered PBS. Samples were incubated with 6.6 μM phalloidin (Invitrogen, cat #A22287, Carlsbad, CA, USA) to stain the actin subcortical cytoskeleton under the plasma membrane, and Hoechst 33,342 (Fisher Scientific, cat #H3570, Waltham, MA, USA) to stain DNA. Samples were placed into an 18-well, #1.5 glass bottom chamber slides (IBIDI, cat #81817, Fitchburg, WI, USA) and imaged using a Nikon A1HD (1024) resonant scanning confocal equipped with a sCMOS camera using a 40 X (Plan Fluor, NA 1.3) objective. Images were analyzed in 3D as described below.

### 4.8. Immunofluorescence

Embryos were collected from KSOM culture dishes at 8, 12, 22, or 32 HPI and placed into 4% PFA in filtered PBS for 20 min at room temperature, followed by three washes with 0.1% Triton X-100 in filtered PBS. Samples were then permeabilized with 0.5% Triton X-100 in filtered PBS for 20 min at RT, followed by three washes with 0.1% Triton X-100 in filtered PBS. This was followed by blocking with 10% normal goat serum (Jackson Immunoresearch, cat # 005-000-121, West Grove, PA, USA) in 0.1% Triton X-100 in filtered PBS for one hour at room temperature, before incubation with primary antibody solutions overnight at 4 °C. Embryos were stained with the following antibodies: anti-H3K4me3 [1:100] (Cell Signaling, cat #9751S, Danvers, MA, USA) and anti-H3K27ac [1:100] (ThermoFisher, cat #39034, Waltham, MA, USA). The following day, samples were washed three times with 0.1% Triton X-100 in filtered PBS, before incubation with antirabbit secondary AlexaFluor-488 [1:100] (Invitrogen, cat #A11008, Waltham, MA, USA) supplemented with of 6.6 μM phalloidin and Hoechst 33,342 (Fisher Scientific, cat #H3570, Waltham, MA, USA). Samples were placed into an 18-well, #1.5 glass bottom chamber slides (IBIDI, cat #81817, Fitchburg, WI, USA) and imaged using a Nikon A1HD (1024) resonant scanning confocal equipped with a sCMOS camera using a 40X (Plan Fluor, NA 1.3) objective.

### 4.9. Imaging Analysis

Analysis was performed using the Nikon Elements Analysis 3.1 software. Semiautomated analyses were performed using the General Analysis 3 (GA3) software. The software allows for a series of binary and image processing tools within individual channels to identify individual identification of objects by combing intensity range, separation, smooth, and object binary functions to isolate and identify single nuclei within an embryo. Following identification, the program calculated general parameters such as pronuclear volume and quantification of signal intensity in each specific channel. Immuno-stained intensity data were normalized dividing the intensity of the post-translational histone modification signal over the intensity of the Hoechst 33342. The pronuclear stage was evaluated in 3D-reconstructed images for a more accurate measurement of the relative distance between male and female PN.

### 4.10. Single Embryo RNAseq

Single embryo RNA-seq libraries were generated according to Smart-seq3 protocol with several modifications [52]. Briefly, individual two-cell embryos were collected in a 1x TCL buffer (Qiagen, cat #1070498, Germantown, MDss) containing 1% beta-mercaptoethanol (Sigma, cat #M6250, St. Louis, MO, USA). Total RNA were purified by 2.2x SPRI beads (Beckman B23318) and the beads were resuspended in 6 µL of lysis buffer (0.5 U/µL of recombinant RNase inhibitor (RRI) (Takara, 2313B, Kusatsu, Shiga, Japan), 0.15% Triton X-100, 0.5 mM dNTP (Thermo Fisher Scientific, R0181, Waltham, MA, USA), 1 µM Smart-seq3 oligo-dT primer (5′-biotin-ACGAGCATCAGCAGCATACGA T30VN-3′; IDT), 5% PEG 8000 (Sigma, P2139, St. Louis, MO, USA)). Samples were incubated at 72 °C for 10 min and on ice afterwards. Next, 2 µL of reverse transcription mix (25 mM Tris-HCL, pH 8.3 (Sigma, T6791, St. Louis, MO, USA), 30 mM NaCl (Ambion, AM9759, Austin, TX, USA), 1 mM GTP (Thermo Fisher Scientific, R0461, Waltham, MA, USA), 2.5 mM MgCl2 (Ambion, AM9530G, Austin, TX, USA), 8 mM DTT (Thermo Fisher Scientific R0861, Waltham, MA, USA), 0.5 U/µL RRI (Takara, 2313B, Kusatsu, Shiga, Japan), 2 µM of different Smart-seq3 TSOs (5′-biotin AGAGACAGATTGCGCAATGNNNNNNNNrGrGrG-3′; IDT), and 2 U/µL of Maxima H-minus reverse transcriptase enzyme (Thermo Fisher Scientific, EP0751, Waltham, MA, USA) were added to each sample. Reverse transcription and template switching were carried out at 42 °C for 90 min, then 10 cycles of 50 °C for 2 min, and 42 °C for 2 min, and 85 °C for 5 min. PCR preamplification was performed by adding 12 µL of PCR mix (1x KAPA HiFi master mix, 0.1 µM Smartseq3 forward PCR primer (5′-TCGTCGGCAGCGTCAGATGTGTATAAGAGACAGATTGCGCAATG-3′; IDT) and 0.1 µM Smartseq3 reverse PCR primer (5′-ACGAGCATCAGCAGCA TACGA-3′; IDT)). PCR was conducted as follows: 3 min at 98 °C, 19 cycles of 20 s at 98 °C, 30 s at 65 °C, 6 min at 72 °C, and 5 min at 72 °C. PCR product was purified by 0.8x SPRI beads and eluted in nuclease-free water (Ambion, AM9932, Austin, TX, USA).

Purified cDNA was quality checked by a bioanalyzer and quantified using Qubit 1X dsDNA HS Assay Kit (Thermo Fisher Scientific, Q33231, Waltham, MA, USA). Sequence libraries were prepared using Illumina Nextera XT DNA Library Prep kit (Illumina, FC-131-1096, San Diego, CA, USA). Libraries were sequenced at paired end (45 bp for read 1 and 30 bp for read 2) 30-bp on an Illumina NextSeq500 instrument.

### 4.11. Analysis of Smart-seq3 Sequencing Data

Raw fastq files were separated into 5′ end UMI-containing reads and internal reads based on the 11-bp tag sequence (ATTGCGCAATG). For the 5′ end reads, UMI sequence was extracted by UMI tools [53], followed by transcriptome alignment using STAR [54]. Only unique mapping reads were kept, and PCR duplicates were removed by UMI tools. For the internal reads, read alignment was performed using STAR and deduplication was carried out by Picard [55]. The resulting bam files from the 5′ end and internal reads were merged. Quantification of read counts was performed by using FeatureCounts [55], and batch-effect correction was performed using Combat-seq [56]. Differential expressed genes were identified using DESeq2 [57], using a multiple hypothesis-adjusted *p* value of 0.1.

### 4.12. Statistical Analysis

Data from all studies were analyzed using GraphPad Prism version 9.3.1 (471) for Windows, GraphPad Software, San Diego, CA, USA, www.graphpad.com. Data are expressed as the mean ± S.E.M with individual experimental values represented by dots. Direct paired control- and SER-generated data points were compared by either nonpaired *T*-tests, or nonparametric, or two-way ANOVA when applicable. Specific details of statistical tests performed are detailed in the figure legends of each graph. Data representing PN staging and NEBD were represented as graphs to demonstrate the frequency distribution and Chi-square tests were performed to analyze statistical differences.

## Figures and Tables

**Table 1:** Timing of nuclear envelope breakdown (NEBD) using time-lapse microscopy. Time-lapse analysis of control- and SER-generated embryos was performed and the timing of NEBD was categorized into four categories: fast (9.0–14.5 HPI), medium type I (15.0–16.0 HPI), medium type II (16.5–17.5 HPI) and slow (18.0+ HPI). Quantification of individual embryo numbers within each category. N = 4 independent experiments.

	Frequency of Embryos (%)			
	Timing of NEBD			
	9.0–14.5 HPI	15.0–16.0 HPI	16.5–17.5 HPI	18.0+ HPI
Control	3/37 (8.1)	8/37 (21.6)	18/37 (48.6)	8/37 (21.6)
SER	5/43 (11.6)	17/43 (39.5)	16/43 (37.2)	5/43 (11.6)

## Data Availability

The accession number for the sequencing data reported in the paper is GEO: GSE221741.

## Supplementary Materials

The following supporting information can be downloaded at: https://www.mdpi.com/article/10.3390/ijms24010640/s1.
