# Supplementary material for: Sperm Energy Restriction and Recovery (SER) Alters Epigenetic Marks during the First Cell Cycle of Development in Mice
Source: Int J Mol Sci. 2022 Dec 30;24(1):640. doi: 10.3390/ijms24010640 (PMC9820464; doi:10.3390/ijms24010640)
Supplement: Supplementary file 1 [file ijms-24-00640-s001.zip › ijms-1969592-supplementary.pdf]

Experiment #	Control			SER		
	Total	Fertilized	%	Total	Fertilized	%
**1**	29	6	20.7	31	29	93.5
**2**	37	0	0.0	60	53	88.3
**3**	90	27	30.0	92	74	80.4
**4**	16	5	31.3	17	12	70.6
**5**	73	29	39.7	88	65	73.9
**6**	65	19	29.2	59	40	67.8
**7**	29	8	27.6	29	20	69.0
**8**	44	25	56.8	59	44	74.6
**9**	69	25	36.2	80	39	48.8
**10**	63	15	23.8	80	31	38.8
**11**	88	34	38.6	70	52	74.3
**12**	91	47	51.6	130	70	53.8
**13**	106	39	36.8	85	38	44.7
**14**	83	45	54.2	73	39	53.4
**15**	77	52	67.5	74	49	66.2
**16**	133	79	59.4	117	99	84.6
**17**	183	81	44.3	172	106	61.6
**18**	98	38	38.8	95	67	70.5
**19**	95	36	37.9	74	50	67.6
**20**	62	29	46.8	70	44	62.9
**21**	200	124	62.0	182	139	76.4
**22**	101	72	71.3	95	84	88.4
**23**	54	24	44.4	91	44	48.4
**24**	33	7	21.2	80	54	67.5
**25**	85	25	29.4	107	66	61.7
**26**	63	16	25.4	17	10	58.8
**27**	85	30	35.3	105	44	41.9
**28**	124	86	69.4	136	106	77.9
**29**	72	34	47.2	63	42	66.7
**30**	105	59	56.2	85	59	69.4
**31**	77	20	26.0	66	41	62.1
**32**	88	57	64.8	71	60	84.5
**33**	104	48	46.2	92	70	76.1
**34**	130	44	33.8	153	93	60.8
**35**	174	72	41.4	133	83	62.4
**Total**	**3026**	**1357**	**41.3**	**3031**	**2016**	**67.1**

Supplemental Table S1 Fertilization rates after IVF. Fertilization was evaluated by visualization of 2-cell embryos 18 HPI with control- or SER-treated sperm. Numbers of total and fertilized eggs in each experiment are displayed.

Control Embryos at 96 HPI							
**Experiment #**	**2-Cell**	**4-Cell**	**8-Cell**	**Morula**	**Early Blastocyst**	**Late Blastocyst**	**Degraded**
**1**	80.0	0.0	0.0	0.0	0.0	20.0	0.0
**2**	29.4	14.7	0.0	2.9	2.9	50.0	0.0
**3**	0.0	33.3	0.0	0.0	0.0	66.7	0.0
**4**	0.0	0.0	0.0	11.8	23.5	64.7	0.0
**5**	8.3	0.0	4.2	8.3	29.2	45.8	4.2
**6**	10.7	14.3	0.0	28.6	10.7	25.0	10.7
**7**	0.0	4.2	0.0	0.0	0.0	66.7	29.2
**8**	0.0	4.0	0.0	0.0	4.0	84.0	8.0
**9**	8.0	0.0	0.0	0.0	0.0	88.0	4.0
**10**	17.6	23.5	0.0	17.6	11.8	29.4	0.0
**11**	10.0	0.0	0.0	5.0	10.0	75.0	0.0
Average (SEM)	14.9 (±21.8)	8.5 (±9.1)	8.5 (±9.1)	6.8 (±3.2)	8.4 (±6.6)	55.9 (±12.4)	5.1 (±0.0)

SER Embryos at 96 HPI							
**Experiment #**	**2-Cell**	**4-Cell**	**8-Cell**	**Morula**	**Early Blastocyst**	**Late Blastocyst**	**Degraded**
**1**	0.0	0.0	0.0	13.8	6.9	72.4	6.9
**2**	0.0	4.1	0.0	2.7	6.8	79.7	6.8
**3**	0.0	9.5	0.0	4.8	0.0	81.0	4.8
**4**	4.0	8.0	0.0	4.0	0.0	84.0	0.0
**5**	6.1	0.0	0.0	0.0	12.1	81.8	0.0
**6**	0.0	12.5	0.0	6.3	6.3	68.8	6.3
**7**	0.0	4.0	0.0	0.0	0.0	96.0	0.0
**8**	0.0	0.0	0.0	0.0	0.0	97.0	3.0
**9**	0.0	3.8	0.0	0.0	0.0	92.3	3.8
**10**	18.5	3.7	3.7	29.6	0.0	44.4	0.0
**11**	0.0	0.0	0.0	0.0	5.9	82.4	11.8
Average (SEM)	2.6 (±1.2)	4.1 (±2.5)	0.3 (±0.0)	5.6 (±2.9)	3.4 (±2.3)	80.0 (±2.8)	3.9 (±1.9)

Supplemental Table S2 Stage of embryonic arrest at 96 HPI. Control- and SER-generated embryos were cultured in vitro after IVF. Embryonic arrest was evaluated after 96 HPI and broken down into 7 categories: 2-cell, 4-cell, 8-cell, morula, early blastocyst, late blastocyst, and degraded. Percentages for each independent experiment is shown.
