# Supplementary figures and images for: Sperm Energy Restriction and Recovery (SER) Alters Epigenetic Marks during the First Cell Cycle of Development in Mice
Source: Int J Mol Sci. 2022 Dec 30;24(1):640. doi: 10.3390/ijms24010640 (PMC9820464; doi:10.3390/ijms24010640)

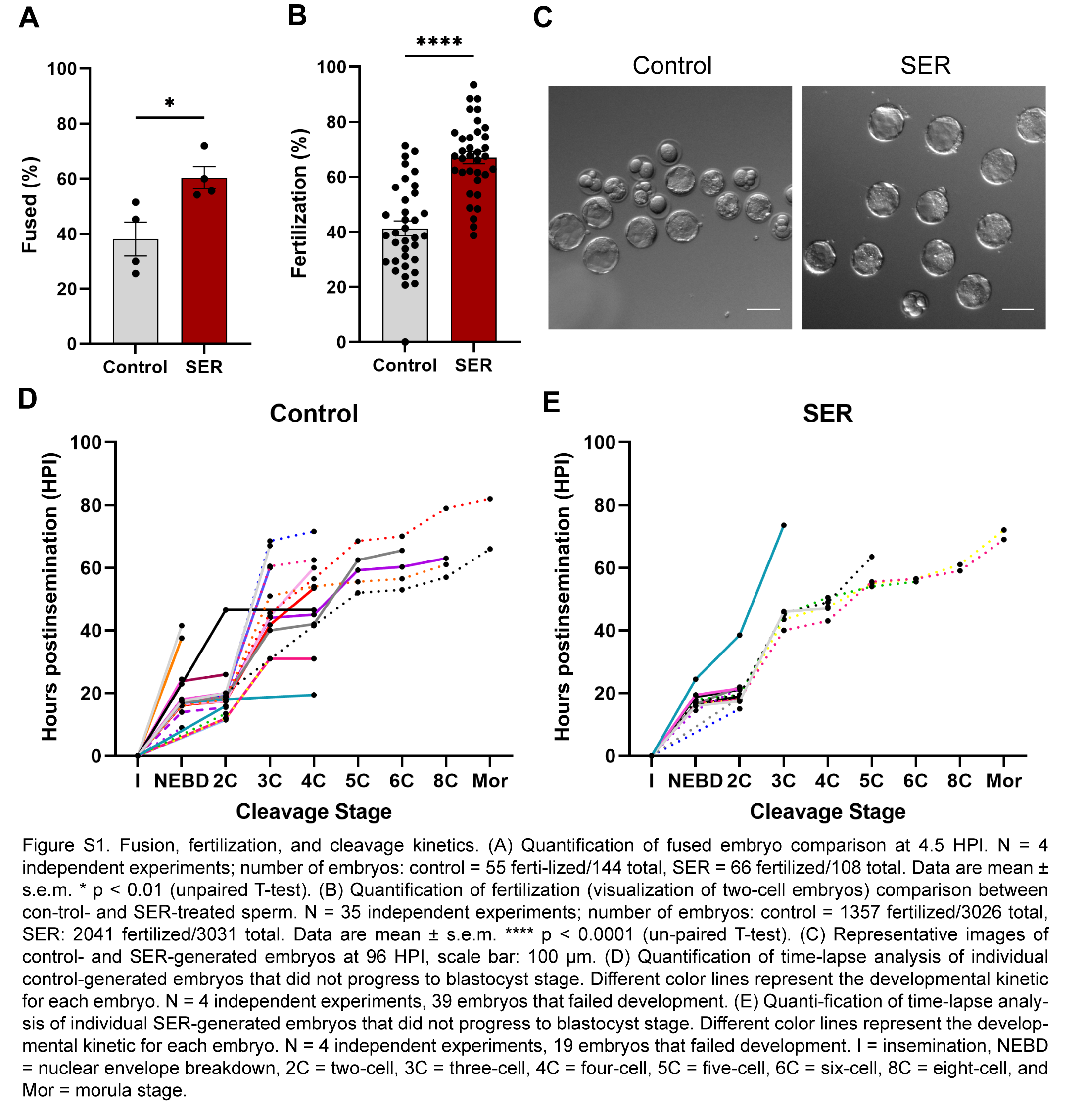

Supplement: Supplementary file 1 [file ijms-24-00640-s001.zip › Tourzani_PN_SFigure 1.tif]
